# Acquisition of Dynamic Material Properties in the Electrohydraulic Forming Process Using Artificial Neural Network

**DOI:** 10.3390/ma12213544

**Published:** 2019-10-29

**Authors:** Min-A Woo, Young-Hoon Moon, Woo-Jin Song, Beom-Soo Kang, Jeong Kim

**Affiliations:** 1 Department of Aerospace Engineering, Pusan National University, Busan 46241, Korea; alsdk0072@pusan.ac.kr (M.-A.W.); bskang@pusan.ac.kr (B.-S.K.); 2 Department of Mechanical Engineering, Pusan National University, Busan 46241, Korea; yhmoon@pusan.ac.kr; 3 Department of Green Transportation System, Graduate School of Convergence Science, Pusan National University, Busan 46241, Korea; woodysong@pusan.ac.kr

**Keywords:** electrohydraulic forming, material property, surrogate model, reduced order model, artificial neural network

## Abstract

Electrohydraulic forming is a high-velocity forming process that deforms sheet metals with velocities above 100 m/s and strain rates more than 100 s^−1^. This experiment was conducted in a closed space because of safety concerns related to the high-velocity conditions; therefore, we were not able to examine the deformation process of the sheet metal. To observe the electrohydraulic forming process in detail, we performed virtual numerical simulations using accurate material properties. Therefore, in this paper, we obtained the material property of a sheet metal from a numerical estimation by using a surrogate model based on the reduced order model and the artificial neural network. The Cowper–Symonds constitutive equation was selected for the Al 6061-T6 sheet metal, and two strain rate parameters were adopted as the unknown parameters. From the two sampling techniques, the training and test samples were extracted from the specific ranges of two unknown parameters, and a numerical simulation was performed for these samples by using the LS-DYNA program. The z-axis displacements of the deformed sheet metal were obtained from the results of the numerical simulation, and two basis vectors were extracted by using principal component analysis. In addition, to predict the weighting coefficients of the two basis vectors at the defined range of parameters, we used the artificial neural network technique as a surrogate model. By comparing the surrogate model and the experimental results and calculating the root mean square error value, we estimated the optimal parameter for Al 6061-T6. Finally, the reliability of the obtained material parameters was proved by comparing the experimental results, the surrogate model, and LS-DYNA.

## 1. Introduction

**Figure 1:**
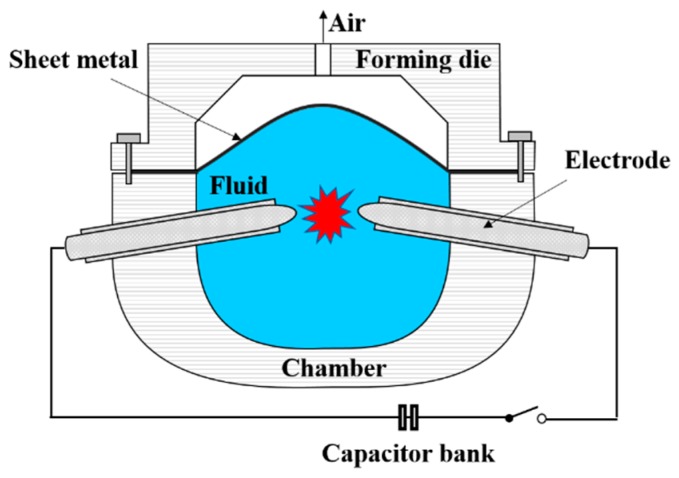
Conceptual diagram for electrohydraulic forming.

Electrohydraulic forming (EHF) is a high-velocity sheet metal forming process that deforms the sheet with a velocity greater than 100 m/s and a strain rate above 100 s^−1^. In particular, EHF uses the discharge of electrical energy in a fluid (e.g., water or oil) as a deformation source. When a capacitor bank discharges electrical energy, the energy is delivered to the fluid by the two electrodes attached to the wall of the chamber, and this discharge creates high-pressure shockwaves. A very thin wire connects the tips of the two electrodes. Therefore, the transmitted energy causes the wire to explode, which maximizes the shockwaves in the fluid. The metal sheet that was in contact with the fluid is deformed into the die by the shockwaves. A schematic view of the EHF process is shown in Figure 1.

Researchers have found that the high-velocity forming process can improve the formability of a sheet metal having low formability, such as magnesium alloys, titanium alloys, and some aluminum alloys [1]. Shim and Kang [2] used the 5000 series of aluminum alloys for the EHF experiment and showed formability improvement in the EHF process by comparing Forming limit diagrams (FLDs) in quasi-static and high-speed conditions. Gillard et al. [3] and Golovashchenko et al. [4,5] showed that the formability of the DP 980 sheet was improved by die–sheet interactions in the EHF experiment using dies of various shapes. 

Besides EHF, electromagnetic forming (EMF), which is based on the use of a magnetic force generated from a copper coil, shows improvements in formability. Imbert [6] showed significant improvements in formability in the EMF experiment and elucidated the reason for the bending and straightening of the sheet metal. In addition, Imbert et al. [7] found that high hydrostatic stresses, which are favorable for suppression of damage and increase in sheet ductility, can increase formability in the EMF process. However, EMF suffers from a major disadvantage—the forming force is affected by the electrical conductivity of the sheet metal. For materials with low electrical conductivity, the forming efficiency is so low that the EMF has limitations in a wide range of industries as described by Shin [8]. Therefore, the EHF, which does not have any restrictions on the material selection, has attracted considerable attention from various industries. EHF has another advantage that the bouncing of the sheet metal, which is generated in the EMF process as described by Noh et al. [9], does not occur because the fluid plays an important role in keeping the forming energy at one side of the deformed sheet metal as shown by Woo et al. [10]. 

In a high-velocity forming process, such as in EHF, the progress of the deformation of the sheet metal cannot be observed during the experiment because the deformation is completed in a very short time of approximately 1 ms and the experiment was performed in a closed space because of safety concerns. Therefore, we required a reliable finite element analysis to study the EHF process. For such a reliable analysis, it is necessary to input accurate material properties for the sheet metal to minimize the error between the experiment and numerical simulation. 

In general, for the acquisition of material properties in quasi-static conditions, the tensile test is employed by using a dog bone-shaped specimen at a strain rate below 1 s^−1^ [11]. A popular measurement method used for high-speed conditions is the Split Hopkinson pressure bar (SHPB) test, which shows a strain rate ranging from 10^2^ to 10^3^ s^−1^. SHPB was developed by Kolsky [12] in 1963. In this test, two cylindrical bars are positioned at each end of a specimen. These bars are impacted by a striker bar, and the elastic strain waves measured by using two strain gauges attached to the bar surfaces. Using the strain waves of the two bars and a theoretical equation for SHPB, we calculated the stress–strain relationship of the specimen. However, the experimental apparatus required for the SHPB test is expensive, and it is not suitable as a method for acquiring the properties of the material to be used for sheet metal forming because the properties are obtained by compression force. 

Therefore, in this study, to acquire the material properties of Al 6061-T6, we applied a numerical estimation using a surrogate model that combined the reduced order model (ROM) and the artificial neural network (ANN) such that the numerical simulation of EHF showed results similar to the experimental results. 

To describe the material properties, we employed the Cowper–Symonds constitutive equation that considers a high strain rate condition. We also used two material parameters in this equation as inputs in the surrogate model.

We selected the z-displacement of the sheet metal as the output for the surrogate model. The deformed configuration of the sheet had a high order; therefore, the ROM technique can help the surrogate model generated by the ANN, reduce the computational complexity, and efficiently predict the outputs. 

In Section 2, we describe the numerical model for the EHF free-bulging test and the 40 samples used in the surrogate model. Section 3 explains the detailed process for the construction of the surrogate model by using the ROM technique and the ANN learning model. Section 4 presents the validation of the developed surrogate model by calculating several error values. Section 5 shows the optimized material parameters for Al 6061-T6. Finally, Section 6 summarizes the results of this paper. 

## 2. Finite Element Method for the Electrohydraulic Forming Process

### 2.1. Numerical Modeling 

**Figure 2:**
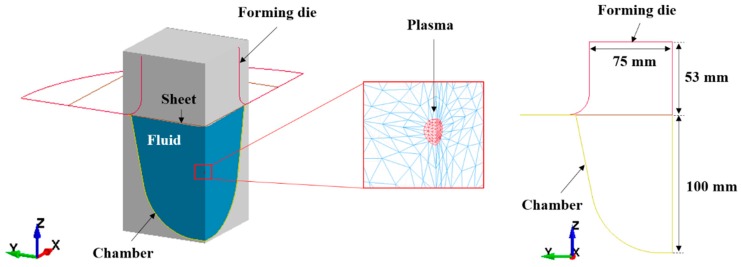
Numerical model for electrohydraulic forming.

To create a surrogate model of EHF, we conducted a numerical simulation by using the LS-DYNA explicit code. The finite element model for the EHF simulation is shown in Figure 2. The fluid parts included plasma, water, and air; the structural parts were composed of a deformable sheet metal, a rigid free-bulging die, and a chamber. To reduce the calculation time, we employed the 1/4 model and applied symmetric conditions in the x–z and y–z planes. 

In the EHF simulation, a large deformation occurs in the fluid parts because of the high electrical energy, which causes a mesh distortion problem. Therefore, in this study, an arbitrary Lagrangian–Eulerian (ALE) method was used to create the elements in the fluid parts. The ALE element can handle the mesh distortion problem, and it is powerful in the fluid-structural coupling problem. Therefore, the ALE element is widely used in many industries, such as automobile, aerospace, and military, which deal with high speeds and large deformation problems [13,14,15]. The structural parts were modeled with the general Lagrangian elements. 

When we define the contact between the ALE materials (plasma, water, and air), we need to merge the nodes located between the different ALE materials and use a multi-material ALE group. For defining the contact condition between ALE and the structural materials, we need to use the constraint keyword. In the constraint keyword, several values should be chosen, such as the number of coupling points, the coupling method, and the penalty coupling force direction. In addition, the slave part for the contact should be the Lagrangian or the structural elements, and the master part should be the ALE or the fluid parts. If the proper parameters are not defined for the constraint keyword, there can be a leakage, which means that the fluids penetrate through the structural parts. Therefore, it is very important to set the parameters for the numerical model. 

In our experiment, we used electrodes to deliver the discharged electrical energy from the capacitor bank to the water. However, in the numerical model, only the plasma part was used instead of the electrodes because of the efficiency of the numerical simulation. If the electrodes were included in the numerical model, the generation of elements in the water part would have become very difficult due to the complex geometry. Therefore, using the plasma part, we describe the pressure wave in the fluid parts.

The electrical power P for the plasma part can be defined by using the current i and the resistance of the wire $R_{w}$ according to the time t in the EHF electrical circuit as described in Equation (1):

(1) $$P=i^{2}R_{w}\left(i,t\right)$$

The time evolution of the current in the EHF process can be defined using the following differential Equation:

(2) $$\frac{d^{2}i}{dt^{2}}+\frac{R_{w}\left(i,\;t\right)+R}{L}\frac{di}{dt}+\frac{1}{LC}i=0$$

Here, i is the current in the circuit, R is the resistance of the circuit except $R_{w}\left(i,t\right)$, C is the capacitance, and L is the inductance. 

However, it is difficult to measure the value of $R_{w}\left(i,t\right)$ because the wire is in the fluid and is dependent on time. Therefore, instead of calculating the current using Equation (2), we used Equation (3) to calculate the electrical power P by measuring the current and voltage (V) curves experimentally as:

(3) P=Vi

**Figure 3:**
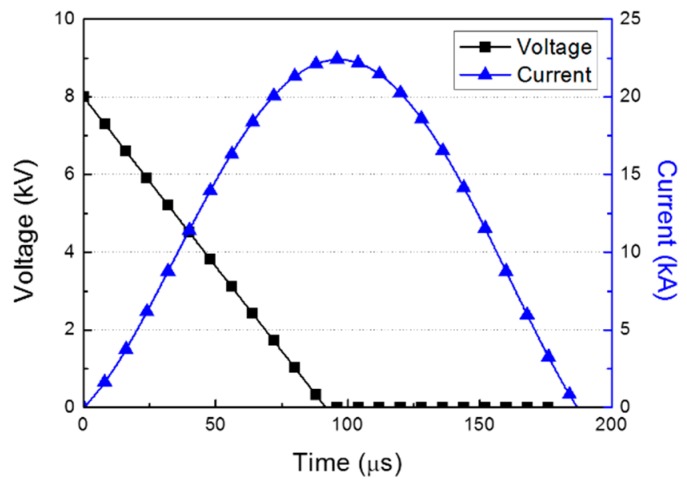
Voltage and current curves in the electrohydraulic forming experiment at 8 kV.

The electrical power for the plasma part was obtained from the EHF experiment for the input voltage of 8 kV. The Rogowski coil surrounded the electric wire, which connected the capacitor bank and the electrodes, and the current was measured when the electrical power was discharged from the capacitor, as shown in Figure 3. The voltage was assumed to decrease linearly from the input to zero when the current was at the maximum point [4]. The electrical power deposition rate obtained by multiplying the current and the voltage curves was the input to the plasma part by using the equation-of-state keyword in LS-DYNA [16]. 

The element size of the fluid parts and the structural parts were set to be like each other so that the deformation energy was delivered stably from the fluid to the structure. 

The thickness of the sheet metal was 1.0 mm, and all the structural parts were constructed with shell elements. The EHF process proceeded under conditions of high strain rate; therefore, we had to choose the material model that considered the flow-stress curves according to the different strain rate conditions for the sheet metal. Therefore, the Cowper–Symonds constitutive equation can be written as follows [17]:

(4) $$\bar{\sigma }=\sigma_{0}\left[1+{\left(\frac{\dot{\varepsilon }}{C}\right)}^{1/p}\right],\sigma_{0}=A+B\varepsilon^{n}$$

where $\sigma_{0}$ is the flow stress in a quasi-static condition, ε is the effective plastic strain, $\dot{\varepsilon }$ is the effective strain rate, and *C* and p are the strain rate parameters. *A*, *B*, and *n* are the material properties obtained from the quasi-static tensile test. In the case of Al 6061-T6, *A* = 291 MPa, *B* = 451 MPa, and *n* = 0.66, which were obtained from the tensile test performed at a strain rate of 0.00067 s^−1^. To generate the training and test samples for the surrogate model of the EHF simulation, we selected the strain rate parameters C and p as inputs. The domains for the parameters were selected as follows: $\left[C,\;p\right]=\left[1000,\;2\right]\;\times \;\left[20000,\;20\right]\;\subset \;{\mathbb{R}}^{2}.$ For the 20 training samples, we used the Latin hypercube sampling (LHS) method, and for the 20 test samples, we employed random sampling. 

LHS is a widely used sampling method developed by McKay et al. [18]. It divides the entire range of each variable into n spaces so that the sample is extracted from the entire sample space. We extracted one sample from each space, but not from the overlapping spaces. The advantage of the LHS is that it distributes the samples evenly within a defined range compared with other sampling techniques; therefore, LHS is suitable for extracting the training samples. By random sampling, we obtained 20 test samples that did not overlap with the training samples. 

**Figure 4:**
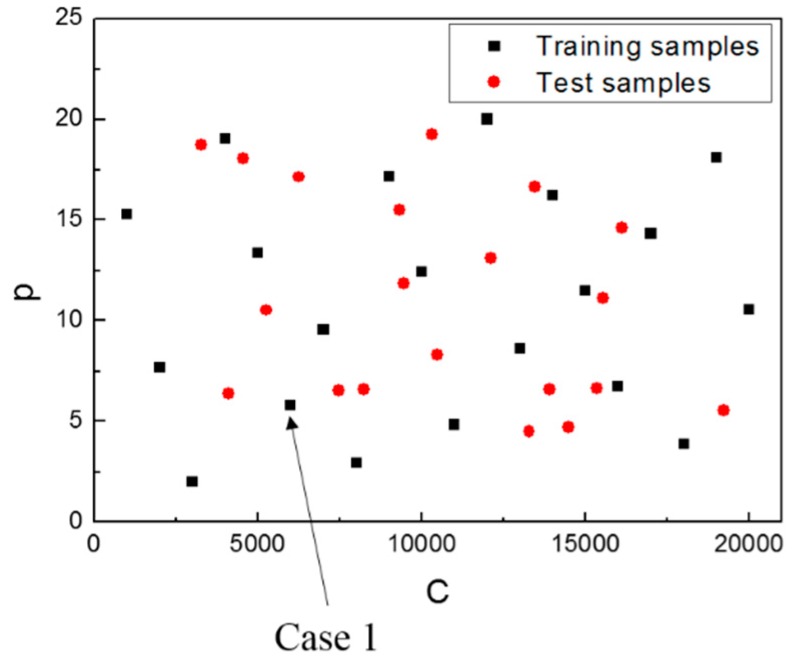
Training and test samples used for the construction of the surrogate model.

The obtained samples are shown in Figure 4. Each parameter sample was input to Equation (4); therefore, we performed a total of 40 numerical simulations. For one case, the analysis required approximately 5 h when the simulation was implemented on a workstation, which was equipped with Intel 3.30 GHz 8 core CPUs and 64 GB RAM. 

The important keywords used in EHF simulation are described in Table 1. 

### 2.2. Numerical Results

**Figure 5:**
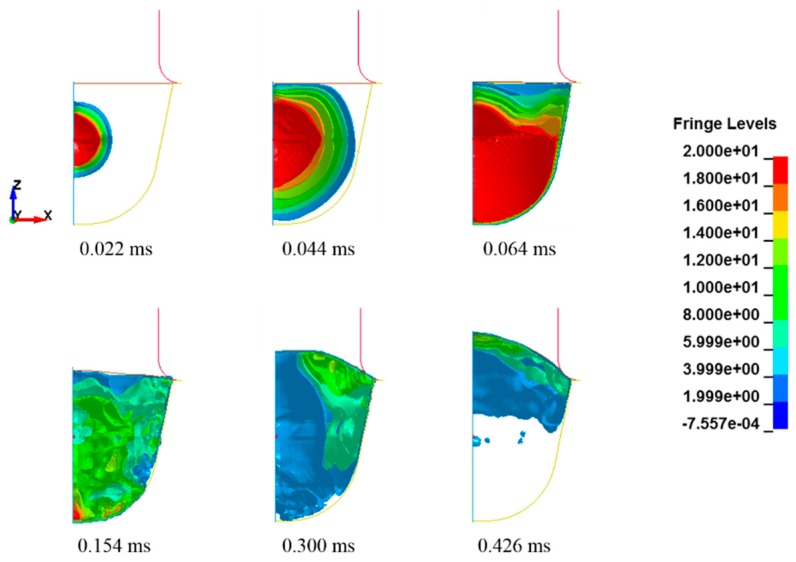
Pressure distribution of the fluid parts in the numerical simulation (unit: MPa).

**Figure 6:**
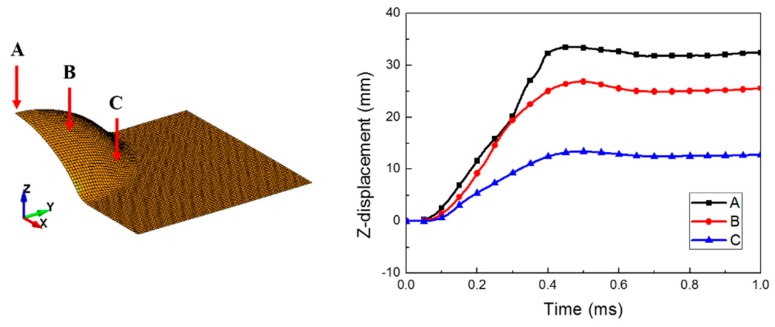
Bulge height of the deformed sheet metal.

The simulation results for case 1 (*C* = 6000 s^−1^ and *p* = 6) are illustrated in Figure 5. The initial plasma part was very small with an initial radius of 1 mm. However, after the power input, the fluid parts expanded, and the water part deformed the sheet metal into the die shape. High pressure first occurred at the center and then propagated throughout the entire fluid parts. However, the energy of the fluid parts was used to deform the sheet metal. Gradually, the pressure decreased, and a slight high pressure was generated near the deformed sheet from 0.3 to 0.4 ms. We could clearly examine the propagation of the pressure waves at the beginning of the power input. However, these waves gradually showed complex shapes due to the interference that occurred as a result of the reflection of the pressure waves against the chamber and the sheet metal. The coupling of the fluid and the structural parts was well defined by using a constraint keyword; therefore, there was no leakage in the numerical simulation. The sheet deformation was almost completed within approximately 0.5 ms; the sheet was saturated until the end of the simulation, as shown in Figure 6. The deformation started at the center of the sheet, and then it proceeded outward.

**Figure 7:**
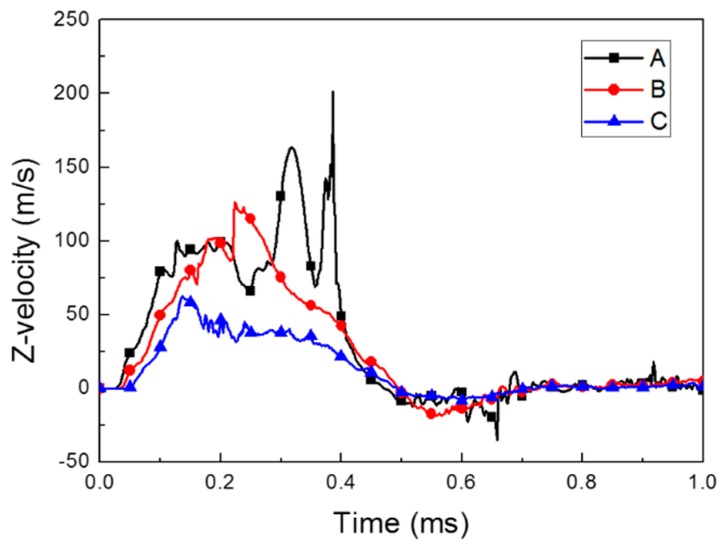
Forming velocity of the deformed sheet metal.

**Figure 8:**
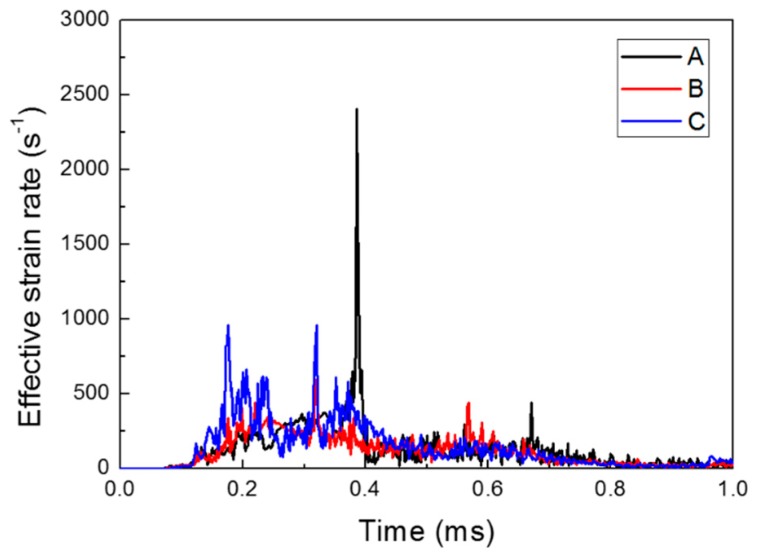
Effective strain rate of the deformed sheet metal.

Figure 7 and Figure 8 show the forming velocity and strain rate for the sheet at three points. As described in the introduction, the sheet metal was deformed with very high velocity above 100 m/s and maximum high strain rates of approximately 2400 s^−1^ at the central area. Mostly, the strain rate of the sheet metal is between 100 and 500 s^−1^ during the forming process. At 0.5 ms, the z-axis velocity had a negative value due to the slight bouncing, which was caused by the vibrations resulting from the high-speed deformation. The z-axis velocity came close to zero as the vibration decreased (see Figure 7). 

**Figure 9:**
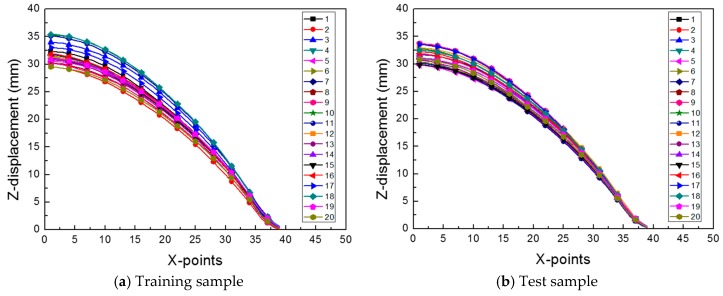
The z-displacement of the sheet metal for (**a**) 20 training samples and (**b**) 20 test samples.

For the 20 training samples and the 20 test samples, the z-displacements of the sheet metal at different x-coordinate points were extracted, as shown in Figure 9. To create the surrogate model, all the cases had the same number of points. Therefore, from the center of the sheet, where the maximum z-displacement occurred, we selected 39 points, and the z-displacement at each point was used in the ROM. 

## 3. Surrogate Model Using Order Reduction and ANN

### 3.1. Reduced Order Model

Reduced order model (ROM) is a mathematical technique used in many fields that deal with high-order numerical models, such as the aviation and aerospace industries [19,20,21]. By using the ROM technique, the original data, which had a high order, was mapped into a lower-dimensional space to reduce the computation time and eliminate unnecessary noise. In this process, the characteristics of the original data are retained. 

To reduce the order of the problem, we performed principal component analysis (PCA) in advance. PCA has been widely used for dimensionality reduction and feature extraction. It finds new basis vectors, which are orthogonal to each other while preserving the variance of the original data and transforming the data from higher-dimensional spaces into lower-dimensional spaces without any linear correlation. 

Let $Z_{c}\left(x;\theta \right)\in {\mathbb{R}}^{39}$ be a mean-centered output associated with the *x*-coordinate ∈ℝ and the material parameter $\theta \left(C,\;p\right)\in {\mathbb{R}}^{2}$ for the training samples. Then, $Z_{c}$ can be presented by the linear combination of the orthogonal basis vectors as follows:

(5) $$Z_{c}\left(x;\theta^{j}\right)=\overset{r}{\underset{i=1}{\sum }}a_{i}\left(\theta^{j}\right)v_{i}\left(x\right)+\;\bar{Z}$$

Here, $a_{i}\left(\theta^{j}\right)$ is the weighting coefficient corresponding to the basis vector $v_{i}\left(x\right)\in {\mathbb{R}}^{m}$, r is the number of basis vectors, $\bar{Z}$ is the sample mean that can be calculated using $\bar{Z}=\;\frac{1}{n}\sum_{j=1}^{n}Z_{c}\left(x;\theta^{j}\right)$ $\in {\mathbb{R}}^{m}$, and n = 20 is the number of samples. 

We can reduce the dimension of the output $Z_{c}$ from r to s by dropping r−s unimportant basis vectors and choosing only the significant s basis vectors. By doing this, Equation (5) can be revised as follows:

(6) $$Z_{c}\left(x;\theta^{j}\right)\approx \overset{s}{\underset{i=1}{\sum }}a_{i}\left(\theta^{j}\right)v_{i}\left(x\right)+\;\bar{Z}$$

We obtained the basis vectors v for this study using the PCA technique as follows. First, using the output of the training sample $Z\in {\mathbb{R}}^{39\;\times \;20}$, we calculated the mean centered sample $Z_{c}$ by subtracting the mean value of the samples from the original data. Second, we determined the eigenvalues and eigenvectors of the covariance matrix of $Z_{c}$. 

When we found the basis vectors of the sample data, we first calculated the covariance matrix of the data. To effectively perform the PCA process, the data Y transformed into the lower-dimensional spaces should preserve the variance of the original data. The transformed data can be described as a linear combination of the original data *X*. Therefore, the transformed new data can be written as $Y=A^{T}X$, where the matrix A is the basis vector. To preserve the variance, the following Equations [22] need to be satisfied by the theory of linear algebra:

(7) $$max\left\{Var\left(Y\right)\right\}=max\{Var(A^{T}X)\}=max\left\{A^{T}\Sigma A\right\}$$

Here, Σ is the covariance matrix of *X*. The value of A is directly proportional to the right-hand-side term of Equation (7). Therefore, using the constraint ||A|| = 1 and applying the Lagrange multiplier method, we obtain the following equation:

(8) (Σ−λ)A=0

From linear algebra [23], we know that A is the eigenvector of Σ, and λ is the eigenvalue of Σ. In conclusion, A (which maximizes the variance of *Y*) is the eigenvector of Σ, and the matrix A is called the PCA. In addition, the column vectors of Σ, which are the eigenvectors, are orthogonal to one another; therefore, they can be used as basis vectors for the linear transformation of X. In conclusion, to find the basis vectors of the data set, we needed to calculate the eigenvectors of the covariance matrix of $Z_{c}$.

**Figure 10:**
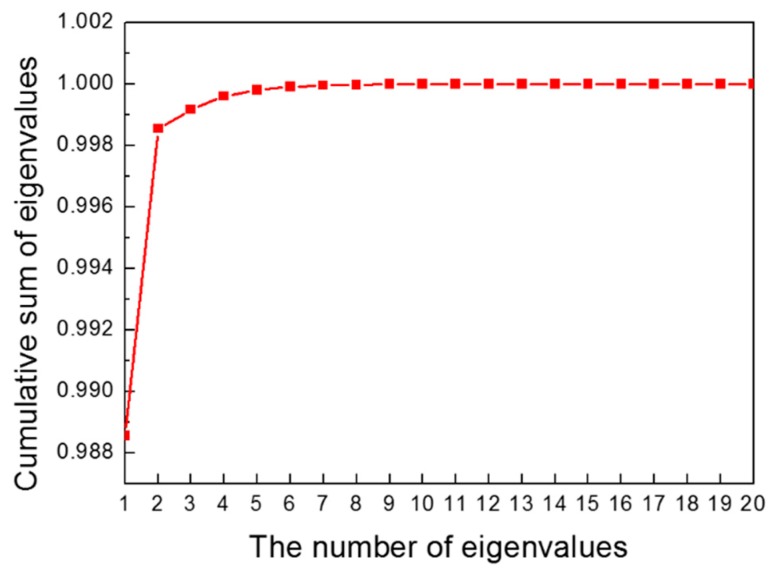
Cumulative sum of eigenvalues.

**Figure 11:**
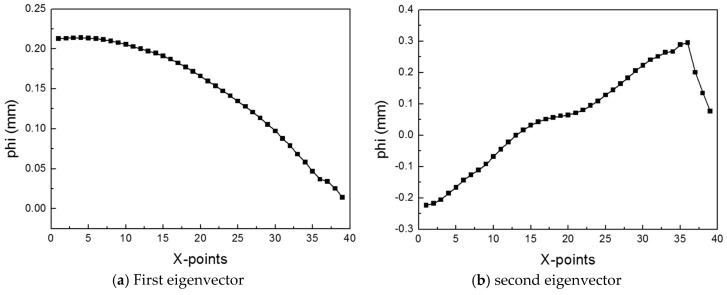
Dominant eigenvectors for the EHF simulation.

In the PCA process, we employed the snapshot method to extract the eigenvalues and eigenvectors. The snapshot method is used to obtain a small number of eigenvectors. When we obtain eigenvalues and eigenvectors, the covariance matrix $Z_{c}Z_{c}{}^{T}$ needs to be calculated. However, for example let A=(m×n) matrix and m≫n, then the size of the covariance matrix $AA^{T}$ increases, and the computation of the eigenvalues and eigenvectors becomes complicated. Therefore, using the snapshot method, we can calculate $A^{T}A$ instead of $AA^{T}$. Using a smaller covariance matrix, we can easily obtain the eigenvalues and eigenvectors. In linear algebra, the eigenvalues of $AA^{T}$ and $A^{T}A$ are the same, and the eigenvectors of $AA^{T}$ can be calculated by multiplying A and the eigenvectors of $A^{T}A$. Therefore, using $Z_{c}{}^{T}Z_{c}$, we computed 20 eigenvalues and 20 eigenvectors, instead of 39 eigenvalues and 39 eigenvectors. We then listed the eigenvectors in the order of magnitude of the eigenvalues. The cumulative sum of the normalized eigenvalues is shown in Figure 10. The sum of all the normalized eigenvalues was 1. The sum of the first and second eigenvalues was 0.9986, which means that the variance of the original data can be preserved more than 99% by using only two eigenvectors. Therefore, the result of the EHF simulation can be represented by using only the first and second eigenvectors. The two eigenvectors are shown in Figure 11. The first eigenvector is similar to the deformation shapes in Figure 9; this means that the first eigenvector is the most important factor in the deformation geometry of the sheet metal and has the same context as the normalized value of the first eigenvalue in Figure 10. Therefore, the first two eigenvectors were chosen as the basis vectors of the ROM. 

### 3.2. Prediction of the Weighting Coefficients by Using ANN

Using the two basis vectors obtained, Equation (6) can be rewritten as follows:

(9) $$Z_{c}\left(x;\theta^{j}\right)\approx \overset{2}{\underset{i=1}{\sum }}a_{i}\left(\theta^{j}\right)v_{i}\left(x\right)+\;\bar{Z}$$

To complete the ROM process, we calculate the weighting coefficients $a_{i}$ at the specific parameter $\theta^{j}$. The weighting coefficient can be easily calculated by orthogonal projection [23]:

(10) $$a_{i}\left(\theta^{j}\right)=\left(Z_{c}{}^{j},\;v_{i}\right).$$

**Figure 12:**
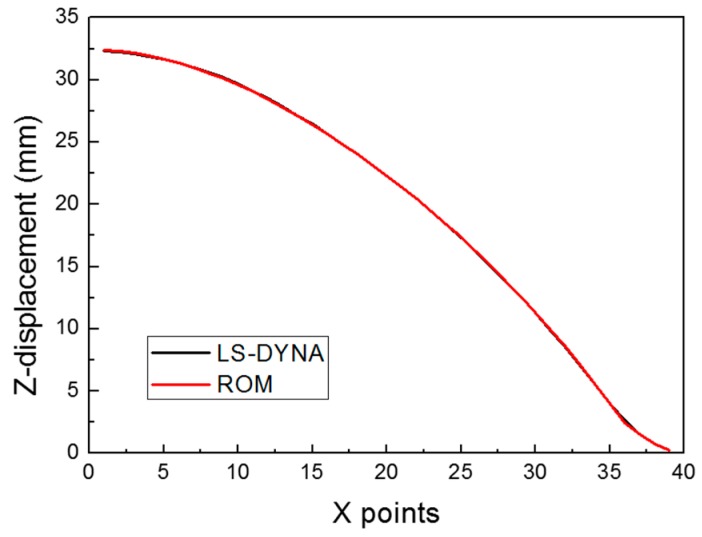
Comparison of LS-DYNA and ROM for Case 1.

For the known parameter $\theta^{j}$, such as the training sample in this study, we can calculate $a_{i}$ because we know the $Z_{c}{}^{j}$ and $v_{i}$ values (which are calculated as described in Section 3.1). For example, for the training sample 1 (C = 6000, p = 5.7895), $a_{1}$ = −1.2490 and $a_{2}$ = 1.4431 from Equation (10). Therefore, when the original z-displacement data is compared with the ROM data, which has only two basis vectors, the two curves show almost the same results as those of the root mean square error of 4.835E−2 (see Figure 12). 

However, for unknown parameters, we cannot obtain $a_{i}$ using Equation (10). Therefore, the ANN model was used to develop a surrogate model that could build the relationship between $\theta^{j}$ and $a_{i}$ such that it was possible to predict $a_{i}$ for the unknown parameter $\theta^{j}$. 

The ANN system is a machine learning method that models the neuronal circuits in which humans make decisions [24]. The ANN system is like the human brain; numerous nodes are interconnected to learn the input data. At the start of the learning, we could not obtain accurate predictions, but the incremental learning created a relationship between the input and output, and the output could be predicted for even untrained input data after the learning was completed.

**Figure 13:**
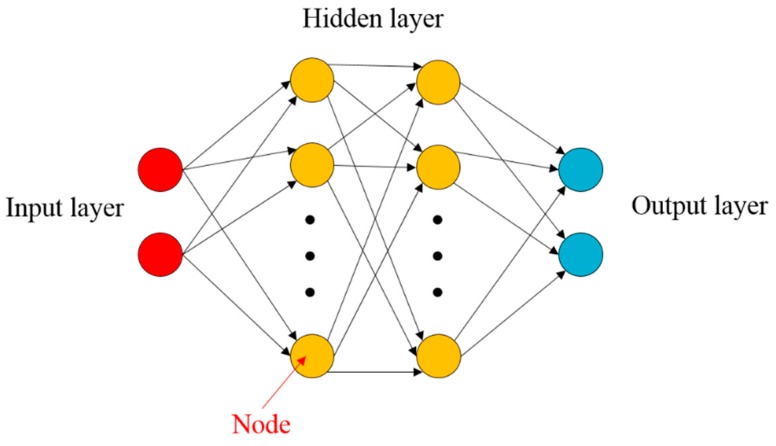
Schematic view of the artificial neural network.

In general, the ANN consists of the input layer, hidden layer, and output layer, as shown in Figure 13. When there are two or more hidden layers, the learning model is known as the deep neural network. The input signal $x_{i}$ is transferred from the input layer to the j-th node of the hidden layer, and it is multiplied by weighting the factor $u_{ij}$. Finally, the input signal is forwarded to the output layer. In this study, we used two hidden layers; therefore, the following equations describe the overall procedures to predict the weighting coefficient in the ROM equation by using ANN:

(11) $$a=\;h\left[\sum^{\text{​}}w_{kl}\;g\left[\sum^{\text{​}}v_{jk}\;f(\sum^{\text{​}}x_{i}u_{ij})\right]\right]$$

Here $u_{ij}$, $v_{jk}$, and $w_{kl}$ are the weight factors from the input to the hidden layer 1, from the hidden layer 1 to the hidden layer 2, and from the hidden layer 2 to the output layer, respectively; f, g, and h are the sigmoid activation functions as follows:

(12) $$f\;=\frac{1}{\left(1+e^{-x}\right)}.$$

The prediction accuracy is improved through a backpropagation algorithm that adjusts the weighting factor between the layers to reduce the error between the output and the desired target values. The Levenberg–Marquardt (LM) function was used as a network backpropagation function [25]. The LM function combines the Gauss–Newton algorithm and the gradient descent algorithm, and it is widely used in nonlinear least-square problems. 

**Figure 14:**
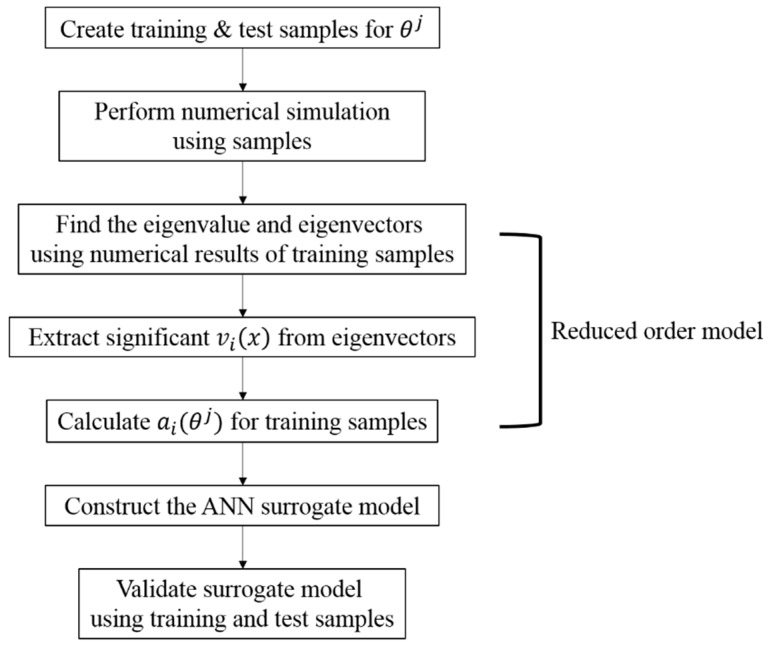
Flowchart for the construction of the surrogate model for the EHF simulation.

In this study, the ANN supervised learning model was constructed using two hidden layers and 40 neurons. In addition, the inputs are the material parameters $\theta^{j}$ for the training samples, and the targets are the weighting coefficient $a_{i}$ corresponding to $\theta^{j}$ calculated using Equation (10). The flowchart for constructing the surrogate model using the ROM is shown in Figure 14. 

## 4. Validation of the Surrogate Model

**Figure 15:**
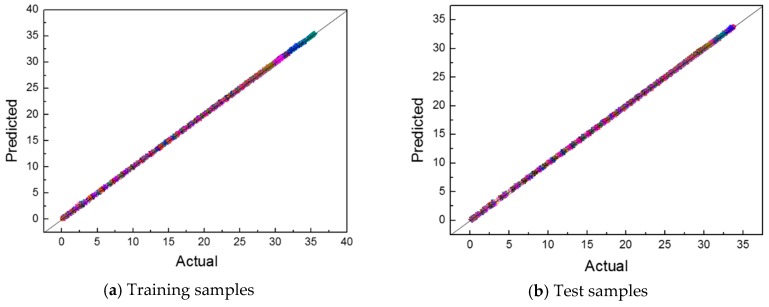
Actual–predicted z-displacement plot for 40 samples. (**a**) Training samples; (**b**) Test samples.

The surrogate model using the ROM for the EHF was constructed by using the MATLAB 2019a program. Using the constructed surrogate model, we predicted the z-displacement in the EHF process by using the training and test samples. The actual versus predicted plots are shown in Figure 15. Almost all the data are located near the Predicted = Actual line, which means that the outputs of the surrogate model were correctly predicted. 

To analyze the results in detail, we calculated several error values, such as the coefficient of determination (*R*^2^), root mean square error (*RMSE*), mean absolute relative error (*MARE*), and maximum absolute relative error (*Max. ARE*) for the training and test samples, as shown in Table 2. A regression model with *R*^2^ close to zero is not very useful; however, when *R*^2^ has a large value close to one, the output is predicted well. The *RMSE* value is commonly used to calculate the difference between the prediction of the regression model and observation of the real experiment. *MARE* is employed in regression problems or model evaluation owing to its intuitive interpretation in terms of the relative error. The equations for the four errors are as follows:

(13) $$R^{2}=\frac{\sum^{\text{​}}{\left({\hat{y}}_{i}-{\bar{y}}_{i}\right)}^{2}}{\sum^{\text{​}}{\left(y_{i}-{\bar{y}}_{i}\right)}^{2}}$$

(14) $$RMSE=\sqrt{\frac{1}{n}\Sigma {\left(y_{i}-{\hat{y}}_{i}\right)}^{2}}$$

(15) $$MARE=\frac{1}{n}\Sigma \left|\frac{y_{i}-{\hat{y}}_{i}}{y_{i}}\right|$$

(16) $$Max.ARE=max\left|\frac{y_{i}-{\hat{y}}_{i}}{y_{i}}\right|$$

Here, $y_{i}$ is the original data, ${\hat{y}}_{i}$ is the predicted value, ${\bar{y}}_{i}$ is the mean value of $y_{i}$, and n is the number of samples. When the *R*^2^ value is close to one, and the *RMSE* and *MARE* values are close to zero, the predicted model estimates very well the output of the original data. 

For the training samples, all the cases show the *R*^2^ values of at least 9.99E−1, and the *RMSE* and *MARE* show values close to zero, and their maximum values were 9.558E−2 and 3.036E−2, respectively. All the *Max. ARE* also show a small value less than 1. For the training samples, this result is natural because the ANN model for the weighting coefficients was trained by using the training samples. Although the error is a bit large compared with the training data, the test sample also has *R*^2^ values of more than 9.99E−2. The other errors were close to zero; therefore, the test samples also showed good prediction, which means that the outputs of the surrogate model were well predicted. Therefore, we can conclude that the surrogate model was well constructed. 

**Figure 16:**
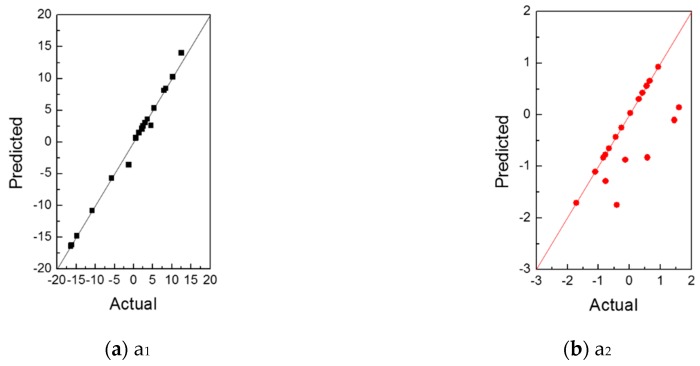
Actual–predicted plot for the weighting coefficients (**a**) a_1_ and (**b**) a_2_.

From the actual–predicted plots for the weighting coefficients shown in Figure 16, we can see that the predicted results of $a_{2}$ have a relatively large error compared with the original values. However, $a_{2}$ is insignificant compared with $a_{1}$ due to the low eigenvalue (described in Section 3.1); therefore, it does not substantially affect the z-displacement results. The results of the surrogate model showed good agreement with the original results notwithstanding the relatively low prediction results of $a_{2}$. 

## 5. Optimal Material Parameters for Al 6061-T6

To find the optimal parameters that have minimum error with the experimental results, we calculated *RMSE* values at the defined range of material parameters as follows:

(17) $$RMSE=\sqrt{\frac{1}{n}\Sigma {\left(y_{experiment}-{\hat{y}}_{estimated}\right)}^{2}}$$

The experimental results were obtained from the free-bulging EHF experiment performed at the input voltage of 8 kV. In the case of the high-velocity forming process, the formability of the sheet metal was affected by the contact between the sheet metal and die [5,6,7]. Therefore, to obtain the accurate material property, the free-bulging die was used so that there was no contact between the sheet metal and the die during deformation. The process for the EHF experiment using the free-bulging die is described in detail in ref. [26]. 

**Figure 17:**
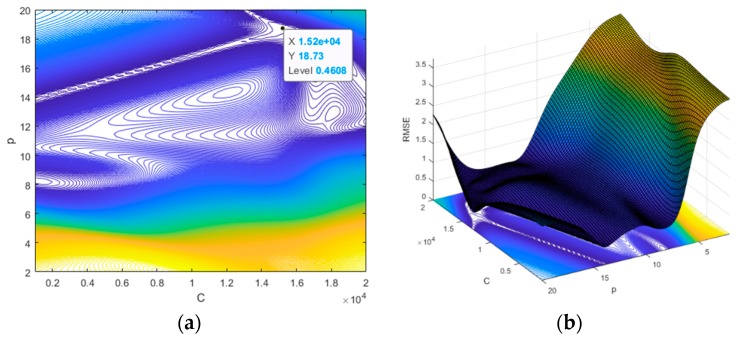
The 2D and 3D plots for the optimized parameters.

**Figure 18:**
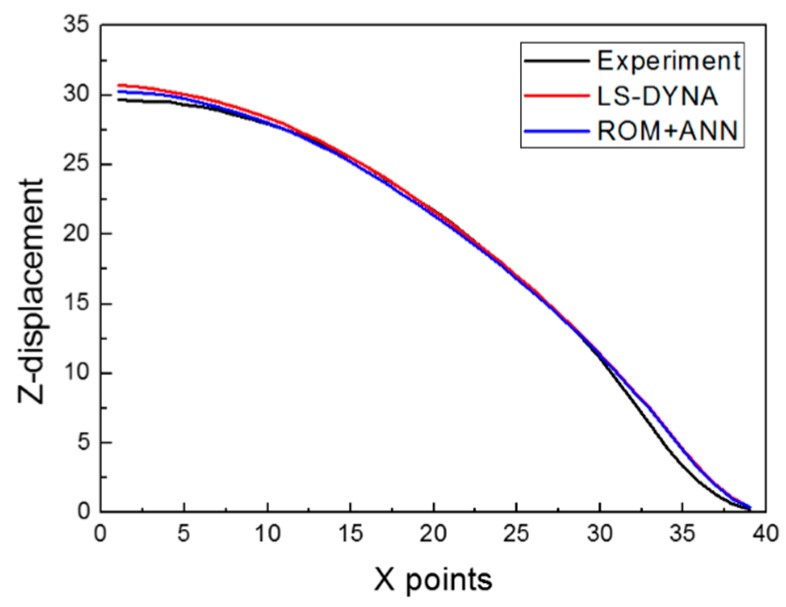
Comparison of the z-displacement in the experiment, the numerical simulation, and the surrogate model at optimized parameters.

The *RMSE* values calculated at $\left[C,\;p\right]=\left[1000,\;2\right]\;\times \;\left[20000,\;20\right]\;\subset \;{\mathbb{R}}^{2}$ are shown in Figure 17 as 2D and 3D graphs by the contour plot function in MATLAB. The minimum value of *RMSE* was approximately 4.608 × 10^−1^, which occurred at C = 15202.02 and p = 18.73. To validate the reliability of the obtained parameters, we compared the following three cases: the surrogate model, the LS-DYNA simulation, and the experiment; our results are shown in Figure 18 and Table 3. A comparison of the numerical simulation–surrogate model shows a higher *R*^2^ value and smaller values of *RMSE*, *MARE*, and *Max. ARE* than the other two cases. This means that the surrogate model, constructed by the ROM and ANN techniques, can well predict the z-displacement in the EHF process. In addition, the other two cases (i.e., the experiment–surrogate model and the experiment–numerical simulation) also show reasonable error values; the R^2^ values were close to 1, and the *RMSE* and *MARE* values were close to 0. Therefore, the material parameters in the Cowper–Symonds model for Al 6061-T6 were determined to be C = 15202.02 and p = 18.73. 

## 6. Conclusions

In this paper, by using the surrogate model based on the ROM and ANN techniques, we performed a numerical estimation to determine the material parameters in the Cowper–Symonds constitutive equation for Al 6061-T6. We obtained 20 training samples and 20 test samples from the LHS and random sampling, respectively, and we conducted numerical simulations for these samples. We extracted z-displacements of the deformed sheet metal and used them to construct the ROM. By applying PCA, we calculated the eigenvalues and eigenvectors for the training samples; two significant eigenvectors were used as the basis vectors in the ROM process. For only two basis vectors, the variance of the original data was preserved over 99%. 

To predict the weighting coefficient for the range of material parameters *C* and *p*, we employed a surrogate model with two hidden layers and 40 nodes. In the ANN model, the input values were the material parameters *C* and *p*, and the output values were the two weighting coefficients in the ROM model having two basis vectors. To perform a goodness of fit for the surrogate model, we calculated *R*^2^, *RMSE*, *MARE*, and *Max. ARE* for the training and test samples. The error showed reasonable values, which proved the reliability of the surrogate model. Finally, we obtained the optimized parameters C = 15202.02 and p = 18.73 by calculating the *RMSE* value for comparing the experimental result and the surrogate model at defined parameter ranges. 

In this study, dynamic material parameters for Al 6061-T6 were obtained using the final shape under the specific conditions of the 8 kV EHF experiment. Since uncertainties, such as input energy error, experimental error, and measurement error, are not considered, the obtained parameters may not be appropriate under other forming conditions. Therefore, in our future work, it will be necessary to use a probabilistic and statistical approach to consider uncertainties to acquire more accurate material parameters which can be applied universally. 

## Figures and Tables

**Table 1:** LS-DYNA keywords used in EHF simulation.

LS-DYNA Keywords	Description
*MAT_PIECWISE_LINEAR_PLASTICITY	Material keyword for Al 6061-T6
*CONTACT_SURFACE_TO_SURFACE	Contact keyword for structural parts
*CONSTRAINED_LAGRANGE_IN_SOLID	Contact keyword for coupling between structure and fluid parts
*INITIAL_VOLUME_FRACTION_GEOMETRY	Volume fraction keyword for generating water part
*EOS_LINEAR_POLYNOMIAL_WITH_ENERGY_LEAK	Equation of state keyword for electric power in plasma part
*ALE_MULTI-MATERIAL_GROUP	Keyword for defining ALE materials group (plasma, water and air)

**Table 2:** Numerical validation for the training and test samples.

Training Sample					Test Smple				
No.	*R* ^2^	*RMSE*	*MARE*	*Max. ARE*	No.	*R* ^2^	*RMSE*	*MARE*	*Max. ARE*
1	9.999E-01	4.835E-02	6.048E-03	8.119E-02	1	9.995E-01	1.983E-01	2.268E-02	2.334E-01
2	9.999E-01	5.312E-02	3.036E-02	7.184E-01	2	9.995E-01	2.189E-01	2.474E-02	2.094E-01
3	9.999E-01	4.087E-02	3.354E-03	3.864E-02	3	9.995E-01	2.082E-01	1.669E-02	1.840E-02
4	9.999E-01	3.374E-02	2.729E-03	2.571E-02	4	9.996E-01	1.987E-01	1.264E-02	5.855E-02
5	9.999E-01	9.558E-02	1.529E-02	3.233E-03	5	9.996E-01	1.992E-01	1.987E-02	2.034E-01
6	9.999E-01	5.749E-02	6.005E-03	1.349E-02	6	9.995E-01	2.122E-01	1.059E-02	1.666E-02
7	9.999E-01	3.348E-02	6.363E-03	2.655E-03	7	9.996E-01	1.974E-01	1.581E-02	1.006E-01
8	9.999E-01	4.260E-02	3.520E-03	2.054E-02	8	9.997E-01	1.981E-01	1.395E-02	6.089E-02
9	9.999E-01	4.228E-02	8.438E-03	1.070E-02	9	9.994E-01	2.033E-01	1.356E-02	1.059E-01
10	9.999E-01	4.052E-02	2.298E-03	6.936E-03	10	9.996E-01	1.977E-01	1.091E-02	3.819E-02
11	9.999E-01	5.653E-02	5.126E-03	5.224E-02	11	9.995E-01	2.092E-01	3.432E-02	4.571E-01
12	9.999E-01	7.672E-02	1.217E-02	7.476E-03	12	9.996E-01	2.007E-01	1.397E-02	6.695E-02
13	9.999E-01	4.057E-02	6.139E-03	3.676E-02	13	9.996E-01	2.059E-01	2.265E-02	1.911E-01
14	9.999E-01	7.306E-02	9.832E-03	9.419E-02	14	9.996E-01	1.999E-01	1.855E-02	1.504E-01
15	9.999E-01	5.273E-02	7.811E-03	7.290E-02	15	9.996E-01	1.986E-01	1.684E-02	1.033E-01
16	9.999E-01	4.708E-02	7.075E-03	6.479E-03	16	9.996E-01	2.152E-01	1.381E-02	3.668E-02
17	9.999E-01	4.191E-02	4.357E-03	2.803E-03	17	9.993E-01	2.045E-01	1.475E-02	8.087E-02
18	9.999E-01	3.483E-02	3.333E-03	2.347E-02	18	9.996E-01	2.012E-01	1.257E-02	5.468E-02
19	9.999E-01	3.159E-02	3.926E-03	3.929E-02	19	9.996E-01	2.019E-01	1.409E-02	8.220E-02
20	9.999E-01	2.188E-02	3.200E-03	5.797E-03	20	9.996E-01	1.976E-01	1.594E-02	9.812E-02

**Table 3:** Numerical validation of the experiment, the numerical simulation, and the surrogate model.

Error	Experiment–Surrogate Model	Experiment–Numerical Simulation	Numerical Simulation–Surrogate Model
*R* ^2^	9.977E-01	9.964E-01	9.985E-01
*RMSE*	4.608E-01	5.793E-01	3.701E-01
*MARE*	6.390E-02	9.620E-02	3.640E-02
*Max. ARE*	3.417E-01	7.980E-01	2.552E-01
